# Prevalence, correlates, and the association of psychotic-like experience with impaired quality of life among Chinese patients with diabetes mellitus: a large-scale cross-sectional study

**DOI:** 10.3389/fpsyt.2025.1563265

**Published:** 2025-10-21

**Authors:** Zhiqi Li, Leiling Liu, Chun Wang, Wanwan Peng, Qiuxia Wu, Pu Peng, Jingjing Zhang

**Affiliations:** ^1^ National Clinical Research Center for Metabolic Diseases, Metabolic Syndrome Research Center, and Department of Metabolism and Endocrinology, The Second Xiangya Hospital of Central South University, Changsha, Hunan, China; ^2^ Department of Cardiovascular Medicine, The Second Xiangya Hospital of Central South University, Changsha, Hunan, China; ^3^ The Fifth Hospital of Jingzhou City, Jingzhou, Hubei, China; ^4^ Jingzhou Central Hospital, Jingzhou, Hubei, China; ^5^ Department of Psychiatry, National Clinical Research Center for Mental Disorders, and National Center for Mental Disorders, The Second Xiangya Hospital of Central South University, Changsha, Hunan, China; ^6^ Department of Psychiatry, Sir Run Run Shaw Hospital, Zhejiang University School of Medicine, Hangzhou, Zhejiang, China

**Keywords:** psychotic-like experiences, diabetes, quality of life, stress, depression

## Abstract

**Introduction:**

While numerous studies have demonstrated a strong association between diabetes mellitus (DM) and psychotic disorders, the relationship between DM and psychotic-like experiences (PLEs)—a subclinical phenomenon at the early stage of the psychosis continuum—remains largely underexplored. This study aimed to investigate the prevalence, correlates, and impact of PLEs on quality of life (QOL) in a large sample of Chinese patients with DM.

**Methods:**

A total of 816 patients with DM and 302 controls were recruited via convenience sampling. PLEs, insomnia, depression, anxiety, stress, diabetes distress, and QOL were assessed using validated questionnaires, alongside demographic and diabetes-related data. Multiple logistic regression models were employed to determine the independent association between DM and PLEs, as well as the correlates of PLEs among patients with DM. Analysis of covariance was used to examine the independent relationship between PLEs and QOL.

**Results:**

The prevalence of any PLEs, frequent PLEs, and clinically relevant PLEs in the DM group was 74.75%, 35.29%, and 13.85%, respectively, compared to 49.67%, 4.30%, and 0.66% in controls. DM was independently associated with higher risks of any PLEs (AOR 1.48, 95% confidence interval (CI) 1.04-2.10), frequent PLEs (AOR 6.56, 95% CI 3.44-12.51), and clinically relevant PLEs (AOR 10.34, 95% CI 2.36-45.35). Age, smoking, diabetes distress, depression, and stress were significant correlates of PLEs. PLEs were significantly associated with lower QOL across all domains.

**Discussion:**

Patients with DM are at a substantially increased risk for PLEs, which significantly impair their QOL. This highlights the need for regular PLEs assessments in routine diabetes care and suggests that interventions targeting depression, diabetes distress, stress, and smoking may help mitigate the burden of PLEs in this population. Future studies should further explore the potential underlying mechanism.

## 1 Introduction

Diabetes mellitus (DM) is a pervasive chronic metabolic disorder with a rapidly increasing global prevalence (1). According to the International Diabetes Federation, over 600 million individuals were living with diabetes worldwide in 2021, and this number is projected to surpass 700 million by 2045 (2). In addition to physical distress, DM is closely associated with mental disorders. A retrospective observational study of 63,365 participants found that 19% of individuals with DM had coexisting mental health disorders, which were linked to a higher mortality risk (odds ratio 1.24; 95% confidence interval 1.16–1.31), particularly in those with schizophrenia (1.82; 95% CI 1.50–2.21) (3).

Schizophrenia is a severe psychiatric disorder characterized by disruptions in thought processes, perceptions, emotional responsiveness, and social interactions (4). Extensive research has elucidated the robust association between schizophrenia and diabetes (5). Cross-sectional studies consistently demonstrate that individuals with schizophrenia have a higher prevalence of diabetes compared to matched control populations (6). Longitudinal cohort studies further support this relationship by revealing that patients with schizophrenia are more likely to develop diabetes over time, even after adjusting for confounding factors such as medication use and lifestyle behaviors (5). Additionally, Mendelian randomization studies provide evidence for a potential causal link between genetic predispositions to schizophrenia and an increased risk of developing diabetes (7), suggesting shared biological mechanisms underpinning both schizophrenia and DM.

Given the robust association between DM and schizophrenia, investigating the risk for psychosis at its earliest stages in this vulnerable population is crucial. The contemporary understanding of psychosis is framed by the ‘extended psychosis phenotype’ model, which posits that psychotic phenomena exist on a dimensional continuum (8, 9). This spectrum ranges from occasional, mild hallucinatory or delusional experiences in the general population at one end, to the frequent and severe symptoms that define clinical psychotic disorders at the other. This model is supported by evidence demonstrating shared genetic, environmental, and neurobiological underpinnings across the full range of expression (10).

Within this framework, psychotic-like experiences (PLEs) are defined as the subclinical manifestations of psychosis that are common in the general population. While not meeting the threshold for a clinical diagnosis, these experiences are highly significant, as they are associated with an increased risk for developing subsequent psychotic disorders, particularly schizophrenia (10–12). Furthermore, even without progressing to a clinical disorder, PLEs are independently associated with a range of negative mental health outcomes, including depression, anxiety, and suicide (13, 14), making them a clinically relevant target for early intervention.

Two large-scale epidemiological studies have provided preliminary evidence suggesting a positive link between DM and PLEs in the general population (15, 16). However, these studies have not delved into the specific prevalence rates of PLEs within the diabetic population or examined the myriad of factors that may contribute to their occurrence. Examining the prevalence and correlates of PLEs in patients with DM—a group with established risk for the most severe end of the psychosis spectrum—offers a critical opportunity to understand the early trajectory of this vulnerability and to identify potential targets for preventive intervention.

Quality of life (QOL) is a multidimensional construct encompassing physical health, psychological well-being, social relationships, and environmental factors (17). In the context of diabetes, QOL serves as a crucial indicator of disease management and overall well-being, as the condition often imposes substantial physical and psychological burdens. In the general population, the presence of PLEs has been consistently associated with reduced QOL, reflecting the detrimental impact of these experiences on daily functioning and mental health (18, 19). However, the specific relationship between PLEs and QOL within the diabetic population remains unclear.

This study aims to address these gaps by conducting a large-scale cross-sectional analysis of Chinese patients with DM. Specifically, the objectives are to: (1) determine the prevalence of any PLEs, frequent PLEs, and clinically relevant PLEs among individuals with DM; (2) identify the correlates associated with PLEs in this population; and (3) examine the impact of PLEs on various domains of QOL. We hypothesize that: (1) Patients with DM are more likely to report PLEs compared to those without DM; (2) DM patients with PLEs demonstrate worse mental health and impaired QOL compared to those without; and (3) PLEs are independently associated with compromised QOL in patients with DM.

## 2 Methods

### 2.1 Study procedure and participants

This hospital-based cross-sectional study was conducted between January 2024 and June 2024. Participants with diabetes mellitus were recruited via convenience sampling from the outpatient and inpatient departments of Jinzhou Hospital, Renmin Hospital of Jinzhou, and the Second XiangYa Hospital. The inclusion criteria were as follows: (1) a confirmed diagnosis of diabetes mellitus as documented in medical records, (2) fluency in Chinese and the ability to comprehend the study questionnaires, and (3) age 18 years or older. Exclusion criteria included the presence of serious physical illnesses, such as organic brain disease or severe infections, a history of dementia, pregnancy, or lactation, and unwillingness to provide informed consent. No restrictions were placed on gender, duration of illness, or type of diabetes.

The control group was recruited through snowball sampling. Specifically, digital posters were created and disseminated via WeChat (China’s largest social media platform) to advertise the recruitment of control participants. We also encouraged those with DM to invite their friends and family members to participate in the survey. Individuals aged 18 years or older who had not been diagnosed with diabetes were eligible to participate in the survey.

Before the survey, research staff provided all participants with a detailed explanation of the study’s objectives, potential benefits, and any associated risks. Participants were informed of their right to withdraw from the study at any time. After obtaining informed consent, participants were asked to complete an electronic questionnaire hosted on WenJuanXin, a widely used online survey platform in China. Research staff were available to clarify any questions the participants had while completing the survey. Upon completion of the questionnaire, participants with diabetes received a gift valued at 5 yuan (approximately $0.70), while control participants received a gift valued at 1 yuan (approximately $0.14). Additionally, participants with diabetes were provided with an electronic version of the “Diabetes Life Guide” as a resource. All participants gave informed consent before the start of the study.

All data collected through the online platform were anonymized; personal identifiers were removed and replaced with a unique study ID to ensure confidentiality. The dataset was stored on a secure, password-protected server (WenJuanXin) accessible only to the primary research team. All study procedures were approved by the Institutional Review Board of the Jingzhou Central Hospital (registration number: 2023-080-01) and adhered to the principles of the Declaration of Helsinki. The differential compensation structure was justified to the IRB by the significantly greater time and effort required from the patient group, who completed a much longer survey than the control group.

### 2.2 Measurements

The online questionnaire comprised both self-designed items and well-established measurement tools, structured into five sections: demographic characteristics, diabetes-related variables, psychotic-like experiences (PLEs), common mental health distress, and quality of life (QOL). Patients with diabetes were required to complete all sections, while healthy controls only responded to the demographic and PLE sections. To ensure data quality, two attention-check questions were embedded in the survey to identify and exclude inattentive participants. The first, “When is the Chinese National Day?”, was a knowledge-based question with a single correct answer, designed to screen out both automated bots and non-conscientious human respondents unfamiliar with the local context. The second, “Have you carefully answered this survey?”, was an instructed-response item intended to capture self-reported engagement. Respondents who answered either of these questions incorrectly were excluded from the final analysis. This dual approach enhances the validity of the collected data by assessing both objective attention and self-attested conscientiousness. These attention-check questions have been widely used in previous epidemiological studies (20, 21).

#### 2.2.1 Demographic information

Demographic data were collected through self-designed questionnaires, capturing the following variables: age, sex (sex generally refers to a set of biological attributes that are associated with physical and physiological features such as chromosomal genotype, hormonal levels, internal and external anatomy) (22), residence (urban/rural), education level (below college/college or above), marital status (single/married), employment status (employed/unemployed), self-perceived economic status (very good/good/average/poor), smoking behavior (current smoker/ex-smoker/non-smoker), drinking behavior (drinker/non-drinker), sleep duration (<4 hours/4–6 hours/6–8 hours/>8 hours), recent weekly exercise (never/sometimes/often/nearly always), and history of psychiatric and physical illnesses.

#### 2.2.2 Diabetes-related variables

We collected the following diabetes-related variables through self-designed questionnaires, including the duration of diabetes, type of diabetes, presence of complications, and current medication plan (insulin therapy/oral hypoglycemic agents/non-pharmacological treatment).

The Chinese version of the Diabetes Distress Scale (DDS) was utilized to assess the emotional burden and distress associated with managing diabetes among the participants (23, 24). It consists of 17 items, each rated on a 6-point Likert scale ranging from 1 (not a problem) to 6 (a very serious problem). The Chinese version of the DDS was widely used in Chinese patients with diabetes (24). Following prior research, a mean score of 3 or above on DDS indicated the presence of severe diabetes distress (24).

#### 2.2.3 Psychotic-like experience

The Chinese version of the 15-item positive subscale of the Community Assessment of Psychic Experiences (CAPE-15) was employed to assess the frequency of psychotic-like experiences over the past month (Sun et al., 2020). The CAPE-15 evaluates symptoms such as delusions, hallucinations, and other positive psychotic symptoms, with responses rated on a 4-point scale from 1 (never) to 4 (nearly always). The total CAPE-15 score ranges from 0 to 45, with higher scores indicating more severe PLEs. In this study, the presence of any PLEs was defined as a score of 1 (“sometimes”) or above on any CAPE-15 item, frequent PLEs were defined as a score of 2 (“often”) or above, and clinically relevant PLEs were defined as a mean CAPE-15 score above 1.20 (25).

#### 2.2.4 Mental health problems

Depressive and anxiety symptoms were assessed through the Chinese version of the 9-item Patient Health Questionnaire (PHQ-9) and the 7-item Generalized Anxiety Disorder Scale (GAD-7) (26, 27). Both scales utilized a 4-point Likert response to assess the frequency of emotional problems within the last week. PHQ-9 and GAD-7 exhibited excellent psychometric properties and were widely used in the Chinese population (28, 29). Following prior research (30, 31), a cutoff of 10 was used to screen for depressive and anxiety symptoms.

We utilized the Chinese version of the Insomnia Severity Index (ISI) to assess insomnia (32). The ISI is a widely used self-report questionnaire consisting of seven items, each rated on a 5-point Likert scale ranging from 0 (no problem) to 4 (very severe problem). The total score ranges from 0 to 28, with higher scores indicating more severe insomnia. The Chinese version of the ISI has been validated in Chinese populations, demonstrating good reliability and validity (32). ISI scores of 8 or above indicated insomnia (33).

The 4-item Perceived Stress Scale (PSS-4) was used to measure the level of perceived stress among participants (34). It is a brief self-report questionnaire designed to assess the degree to which situations in one’s life are appraised as stressful. Each of the four items is rated on a 5-point Likert scale ranging from 0 (never) to 4 (very often), reflecting the frequency of feelings and thoughts related to stress experienced in the past month. The total score ranges from 0 to 16, with higher scores indicating higher levels of perceived stress.

#### 2.2.5 Quality of life

The WHOQOL-BREF, a short form of the World Health Organization Quality of Life (WHOQOL-100) assessment, was used to measure the quality of life (QOL) among patients with diabetes (35). It comprises 26 items, which are divided into four domains: Physical Health, Psychological Health, Social Relationships, and Environment. Each item is rated on a 5-point Likert scale, with higher scores indicating better quality of life. The raw domain scores are transformed to a scale from 0 to 100, and the average scores are calculated to indicate the overall QOL.

### 2.3 Statistical analysis

All statistical analyses were performed on R (ver 4.20). Tests were 2-tailed, and the p-value was set at 0.05 to indicate statistical significance.

First, we conducted descriptive analyses. Assumptions for parametric tests were assessed prior to analysis. The Kolmogorov-Smirnov test indicated that several continuous variables were not normally distributed. Continuous data were presented as medians and interquartile ranges (1st quartile, 3rd quartile), while categorical data were displayed as frequency and percentage. We also conducted Harman’s single-factor test via SPSS and found no common method bias.

Second, we compared the differences in PLEs and demographic information between participants with and without diabetes. Mann-Whitney U test and chi-square tests were performed as appropriate. To avoid false positives, Bonferroni correction was employed, with a corrected p-value of 0.0027 (0.05/18) indicating statistical significance. Multiple logistic regression was used to test whether diabetes was independently associated with PLEs after adjusting for other demographic variables.

Third, we further compared the characteristics of those with or without clinically relevant PLEs in the diabetes groups. Intergroup differences were compared between these two groups, and Bonferroni correction was applied for multiple comparisons (corrected p-value=0.0018). We conducted a multiple logistic regression model using the presence of clinically relevant PLEs as the outcome and other variables exhibiting statistical significance in the univariate analysis as predictors, with an exception of QOL and its domains.

Finally, we performed linear regression model using the QOL and its domains as the outcome and the CAPE-15 scores as the predictor. The model was adjusted for potential confounding effects of sociodemographic and clinical variables, including categorical variables (sex, residence, education level, marital status, employment status, economic status, smoking, drinking, sleep duration, exercise, history of psychiatric and physical illness, type of diabetes, complications, and current medication plan) and continuous variables (age, illness duration, and the scores of PHQ-9, GAD-7, ISI, DDS-17, and PSS).

## 3 Results

### 3.1 Difference in PLEs between patients with and without diabetes mellitus

A total of 1,118 participants were recruited, comprising 816 individuals with diabetes mellitus (DM) and 302 controls. **Table 1** presents the demographic characteristics of the two groups. Compared to controls, patients with DM had significantly higher CAPE-15 scores. The prevalence of any PLEs (74.75% vs. 49.67%), frequent PLEs (35.29% vs. 4.30%), and clinically relevant PLEs (13.85% vs. 0.66%) was substantially higher in the DM group. After adjusting for demographic variables, the presence of DM was independently associated with a higher risk of any PLEs (adjusted odds ratio [AOR] 1.48, 95% confidence interval [CI] 1.04-2.10, *p=*0.028), frequent PLEs (AOR 6.56, 95% CI 3.44-12.51, *p<*0.001), and clinically relevant PLEs (AOR 10.34, 95% CI 2.36-45.35, *p* = 0.002).

**Table 1:** Basic demographic information and psychotic-like experience among participants with and without diabetes mellitus.

Variables	Total (n = 1118)	Without DM (n = 302)	With DM (n = 816)	Statistic	*P*
Age, year M (Q_1_, Q_3_)	53 (36, 63)	52 (47, 60)	54 (34, 65)	Z=-0.07	0.940
Cape15, M (Q_1_, Q_3_)	**2 (0, 6)**	**0 (0, 1)**	**3 (0, 8)**	**Z=-12.32**	**<0.001**
Sex, n(%)				χ²=7.44	0.006
Female	547 (48.93)	168 (55.63)	379 (46.45)		
Male	571 (51.07)	134 (44.37)	437 (53.55)		
Sleep duration, n(%)				**χ²=118.03**	**<0.001**
< 4 hours	136 (12.16)	1 (0.33)	135 (16.54)		
>8 hours	100 (8.94)	12 (3.97)	88 (10.78)		
4–6 hours	445 (39.80)	102 (33.77)	343 (42.03)		
7–8 hours	437 (39.09)	187 (61.92)	250 (30.64)		
Residence, n(%)				χ²=7.26	0.007
Country	473 (42.31)	108 (35.76)	365 (44.73)		
City	645 (57.69)	194 (64.24)	451 (55.27)		
Education, n(%)				χ²=4.51	0.034
Below college	697 (62.34)	173 (57.28)	524 (64.22)		
College or above	421 (37.66)	129 (42.72)	292 (35.78)		
Married status, n(%)				χ²=7.53	0.006
Single	312 (27.91)	66 (21.85)	246 (30.15)		
Married	806 (72.09)	236 (78.15)	570 (69.85)		
Employment status, n(%)				χ²=0.00	0.959
Unemployed	306 (27.37)	83 (27.48)	223 (27.33)		
Employed	812 (72.63)	219 (72.52)	593 (72.67)		
History of psychiatric disorder, n(%)				**χ²=138.76**	**<0.001**
Without	775 (69.32)	290 (96.03)	485 (59.44)		
With	343 (30.68)	12 (3.97)	331 (40.56)		
Economic status, n(%)				**χ²=55.68**	**<0.001**
Very good	253 (22.63)	111 (36.75)	142 (17.40)		
Good	566 (50.63)	139 (46.03)	427 (52.33)		
Normal	252 (22.54)	49 (16.23)	203 (24.88)		
Bad	47 (4.20)	3 (0.99)	44 (5.39)		
Smoking status, n(%)				**χ²=35.14**	**<0.001**
Non-smoker	758 (67.80)	235 (77.81)	523 (64.09)		
Ex-smoker	124 (11.09)	7 (2.32)	117 (14.34)		
Current smoker	236 (21.11)	60 (19.87)	176 (21.57)		
Drinking behavior, n(%)				χ²=0.21	0.646
Non-drinker	675 (60.38)	179 (59.27)	496 (60.78)		
Drinker	443 (39.62)	123 (40.73)	320 (39.22)		
Exercise, n(%)				**χ²=42.93**	**<0.001**
Never	80 (7.16)	17 (5.63)	63 (7.72)		
Sometimes	366 (32.74)	61 (20.20)	305 (37.38)		
Often	311 (27.82)	120 (39.74)	191 (23.41)		
Nearly	361 (32.29)	104 (34.44)	257 (31.50)		
History of physical illness, n(%)				**χ²=90.59**	**<0.001**
0	384 (34.35)	163 (53.97)	221 (27.08)		
1-2	613 (54.83)	136 (45.03)	477 (58.46)		
>3	121 (10.82)	3 (0.99)	118 (14.46)		
With any PLEs, n(%)				**χ²=63.72**	**<0.001**
No	358 (32.02)	152 (50.33)	206 (25.25)		
Yes	760 (67.98)	150 (49.67)	610 (74.75)		
With frequent PLEs, n(%)				**χ²=107.59**	**<0.001**
No	817 (73.08)	289 (95.70)	528 (64.71)		
Yes	301 (26.92)	13 (4.30)	288 (35.29)		
With clinically relevent PLEs, n(%)				**χ²=41.53**	**<0.001**
No	1003 (89.71)	300 (99.34)	703 (86.15)		
Yes	115 (10.29)	2 (0.66)	113 (13.85)		

χ²: Chi-square test Z: Mann-Whitney test

M: Median, Q_1_: 1st Quartile, Q_3_: 3st Quartile

Bold suggests statistical significance after multiple correction.

### 3.2 Difference between diabetes patients with and without clinically relevant PLEs

Significant differences were observed between diabetes patients with and without clinically relevant PLEs (**Table 2**). Patients with PLEs were younger, predominantly male (69.03% vs. 51.07%), more likely to be single (52.21% vs. 26.60%), and had higher educational attainment (69.03% vs. 30.44%) (all *p<*0.05). They were also more likely to have a history of psychiatric illness (61.06% vs. 37.27%, *p* < 0.05). Significant differences in lifestyle factors, including sleep duration, smoking behavior, drinking behavior, and exercise, were also noted between the groups. However, no significant differences in other demographic variables remained after multiple corrections.

**Table 2:** Comparison between diabetes patients with and without clinically relevant PLEs.

Variables	Total (n = 816)	Without clinically relevant PLEs (n = 703)	With clinically relevant PLEs (n = 113)	Statistic	*P*
*Demographic information*					
Age, M (Q_1_, Q_3_)	**54 (34, 65)**	**57 (38, 66)**	**31 (25, 40)**	**Z=-9.94**	**<0.001**
Sex, n(%)				**χ²=12.62**	**<0.001**
Female	379 (46.45)	344 (48.93)	35 (30.97)		
Male	437 (53.55)	359 (51.07)	78 (69.03)		
Sleep duration n(%)				**χ²=19.56**	**<0.001**
< 4 hours	135 (16.54)	129 (18.35)	6 (5.31)		
4–6 hours	343 (42.03)	300 (42.67)	43 (38.05)		
7–8 hours	250 (30.64)	199 (28.31)	51 (45.13)		
>8 hours	88 (10.78)	75 (10.67)	13 (11.50)		
Residence, n(%)				χ²=0.52	0.470
Country	365 (44.73)	318 (45.23)	47 (41.59)		
City	451 (55.27)	385 (54.77)	66 (58.41)		
Education, n(%)				**χ²=63.08**	**<0.001**
Below college	524 (64.22)	489 (69.56)	35 (30.97)		
College or above	292 (35.78)	214 (30.44)	78 (69.03)		
Married status, n(%)				**χ²=30.33**	**<0.001**
Single	246 (30.15)	187 (26.60)	59 (52.21)		
Married	570 (69.85)	516 (73.40)	54 (47.79)		
Employment status, n(%)				χ²=9.97	0.002
Unemployed	223 (27.33)	206 (29.30)	17 (15.04)		
Employed	593 (72.67)	497 (70.70)	96 (84.96)		
History of psychiatric disorder, n(%)				**χ²=22.86**	**<0.001**
Without	485 (59.44)	441 (62.73)	44 (38.94)		
With	331 (40.56)	262 (37.27)	69 (61.06)		
Economic status, n(%)				χ²=2.42	0.490
Very good	142 (17.40)	118 (16.79)	24 (21.24)		
Good	427 (52.33)	374 (53.20)	53 (46.90)		
Normal	203 (24.88)	172 (24.47)	31 (27.43)		
Bad	44 (5.39)	39 (5.55)	5 (4.42)		
Smoking status, n(%)				**χ²=37.65**	**<0.001**
Non-smoker	523 (64.09)	478 (67.99)	45 (39.82)		
Ex-smoker	117 (14.34)	84 (11.95)	33 (29.20)		
Current smoker	176 (21.57)	141 (20.06)	35 (30.97)		
Drinking behavior, n(%)				**χ²=61.20**	**<0.001**
Non-drinker	496 (60.78)	465 (66.15)	31 (27.43)		
Drinker	320 (39.22)	238 (33.85)	82 (72.57)		
Exercise, n(%)				**χ²=38.44**	**<0.001**
Never	63 (7.72)	53 (7.54)	10 (8.85)		
Sometimes	305 (37.38)	237 (33.71)	68 (60.18)		
Often	191 (23.41)	167 (23.76)	24 (21.24)		
Nearly	257 (31.50)	246 (34.99)	11 (9.73)		
History of physical illness, n(%)				χ²=5.07	0.079
0	221 (27.08)	200 (28.45)	21 (18.58)		
1-2	477 (58.46)	405 (57.61)	72 (63.72)		
>3	118 (14.46)	98 (13.94)	20 (17.70)		
*Diabetes-related variables*					
Duration, M (Q_1_, Q_3_)	5 (2, 11)	5 (2, 11)	3 (2, 10)	Z=-0.22	0.824
Types Of Diabetes, n(%)				**χ²=22.04**	**<0.001**
Type 2	612 (75.00)	547 (77.81)	65 (57.52)		
Type 1	172 (21.08)	133 (18.92)	39 (34.51)		
Others	32 (3.92)	23 (3.27)	9 (7.96)		
Current medication plan, n(%)				χ²=3.69	0.297
oral hypoglycemic agents	354 (43.38)	304 (43.24)	50 (44.25)		
insulin therapy and oral hypoglycemic agents	234 (28.68)	195 (27.74)	39 (34.51)		
insulin therapy	209 (25.61)	187 (26.60)	22 (19.47)		
non-pharmacological treatment	19 (2.33)	17 (2.42)	2 (1.77)		
Complication, n(%)				χ²=6.59	0.010
Without	328 (40.20)	295 (41.96)	33 (29.20)		
With	488 (59.80)	408 (58.04)	80 (70.80)		
DD17, M (Q_1_, Q_3_)	**39 (27, 54)**	**36 (25, 47)**	**69 (63, 76)**	**Z=-15.01**	**<0.001**
Diabetes Distress, n(%)				**χ²=238.56**	**<0.001**
Without	570 (69.85)	561 (79.80)	9 (7.96)		
With	246 (30.15)	142 (20.20)	104 (92.04)		
*Mental distress*					
GAD7, M (Q_1_, Q_3_)	**7 (3, 12)**	**7 (2, 10)**	**12 (10, 15)**	**Z=-11.25**	**<0.001**
Anxiety, n(%)				**χ²=156.26**	**<0.001**
Without	543 (66.54)	526 (74.82)	17 (15.04)		
With	273 (33.46)	177 (25.18)	96 (84.96)		
ISI, M (Q_1_, Q_3_)	**12 (6, 18.25)**	**10 (5, 19)**	**15 (13, 18)**	**Z=-5.47**	**<0.001**
Insomnia, n(%)				**χ²=60.46**	**<0.001**
Without	286 (35.05)	283 (40.26)	3 (2.65)		
With	530 (64.95)	420 (59.74)	110 (97.35)		
PHQ9, M (Q_1_, Q_3_)	**8 (4, 13)**	**7 (4, 11)**	**16 (14, 19)**	**Z=-13.16**	**<0.001**
Depression, n(%)				**χ²=134.02**	**<0.001**
Without	466 (57.11)	458 (65.15)	8 (7.08)		
With	350 (42.89)	245 (34.85)	105 (92.92)		
PSS, M (Q_1_, Q_3_)	**10 (8, 12)**	**10 (8, 12)**	**12 (11, 14)**	**Z=-10.03**	**<0.001**
*Quality of life*					
Overall QOL, M (Q_1_, Q_3_)	**58.61 (49.26, 67.41)**	**60.60 (49.89, 68.43)**	**53.72 (47.88, 58.85)**	**Z=-5.36**	**<0.001**
Physical Health, M (Q_1_, Q_3_)	**57.14 (46.43, 67.86)**	**57.14 (46.43, 71.43)**	**53.57 (46.43, 60.71)**	**Z=-4.21**	**<0.001**
Psychological Health, M (Q_1_, Q_3_)	**50.00 (41.67, 62.50)**	**54.17 (41.67, 66.67)**	**45.83 (37.50, 54.17)**	**Z=-5.69**	**<0.001**
Social Relationships, M (Q_1_, Q_3_)	**66.67 (50.00, 75.00)**	**66.67 (50.00, 75.00)**	**50.00 (41.67, 66.67)**	**Z=-4.43**	**<0.001**
Environment, M (Q_1_, Q_3_)	62.50 (50.00, 68.75)	62.50 (50.00, 68.75)	59.38 (53.12, 62.50)	Z=-2.78	0.005

χ²: Chi-square test Z: Mann-Whitney test

M: Median, Q_1_: 1st Quartile, Q_3_: 3st Quartile

Bold variable indicates statistical significance after multiple correction.

Compared to the non-PLE group, the PLE group had a lower prevalence of type 2 diabetes (57.52% vs. 77.81%, *p* < 0.05) and significantly higher levels of diabetes distress (92.04% vs. 20.20%, *p* < 0.05). Although a higher rate of complications was observed in the PLE group (70.80% vs. 58.04%), this difference did not remain significant after Bonferroni correction. No significant differences were found in illness duration or current medication plans between the groups.

The PLE group exhibited significantly worse mental health, with higher scores on the PHQ-9, GAD-7, PSS, and ISI scales. The prevalence of anxiety (84.96% vs. 25.18%), insomnia (97.35% vs. 59.74%), and depression (92.92% vs. 34.85%) was notably higher in this group. Quality of life was significantly lower in the PLE group across several domains, including overall QOL, physical health, psychological health, and social relationships (all *p* < 0.05). A similar trend was observed in the environmental domain of QOL, although it was not significant after correction.

### 3.3 Independent correlates of PLEs

**Table 3** presents the results of the multiple logistic regression analysis for clinically relevant PLEs. Variables that showed statistical significance in the univariate analysis were included as predictors: age, sex, education level, employment status, marital status, history of psychiatric illness, smoking behavior, drinking behavior, exercise, type of diabetes mellitus, complications, DDS scores, PHQ-9 scores, GAD-7 scores, ISI scores, and PSS scores. In the final model, age (AOR 0.96, 95% CI 0.92-0.99, *p* = 0.037), current smoking status (AOR 2.95, 95% CI 1.06-8.19, *p* = 0.038), DDS scores (AOR 1.09, 95% CI 1.06-1.13, *p<*0.001), PHQ-9 scores (AOR 1.15, 95% CI 1.02-1.29, *p=*0.024), and PSS scores (AOR 1.58, 95% CI 1.30-1.92, *p<*0.001) were independently associated with PLEs.

**Table 3:** Independent correlates of clinically relevant PLEs among patients with DM.

Variables	*P*	OR (95%CI)
Smoking		
Non-smoker		1.00 (Reference)
Ex-smoker	0.072	2.85 (0.91 ~ 8.94)
Current smoker	0.038	2.95 (1.06 ~ 8.19)
Age	0.037	0.96 (0.92 ~ 0.99)
DDS scores	<0.001	1.09 (1.06 ~ 1.13)
PHQ9 scores	0.024	1.15 (1.02 ~ 1.29)
PSS scores	<0.001	1.58 (1.30 ~ 1.92)

OR, Odds Ratio; CI, Confidence Interval

Variables included in the model: age, sex, education level, employment status, marital status, history of psychiatric illness, smoking behavior, drinking behavior, exercise, type of diabetes mellitus, complications, DDS scores, PHQ-9 scores, GAD-7 scores, ISI scores, and PSS scores.

### 3.4 Association between clinically relevant PLEs and impaired quality of life

The results of the linear regression analyses are presented in **Table 4**. In the unadjusted models, PLEs were significantly associated with lower scores across all measured domains of QOL (all p < 0.001). After adjusting for a comprehensive set of demographic, clinical, and psychiatric confounders, this negative association remained statistically significant for overall QOL (β = -0.24, p < 0.001), physical health (β = -0.31, p < 0.001), psychological health (β = -0.20, p = 0.022), and social relationships (β = -0.29, p = 0.005). However, the association with the entertainment domain was attenuated and no longer statistically significant after adjustment (p = 0.054).

**Table 4:** Association of PLEs with QOL among patients with DM.

Outcome: QOL	p-value^a^	β (95%CI)^a^	p-value^b^	β (95%CI)^b^
Overall OQL	<0.001	-0.45 (-0.54 ~ -0.36)	<0.001	-0.24 (-0.36 ~ -0.11)
Physical	<0.001	-0.46 (-0.58 ~ -0.35)	<0.001	-0.31 (-0.45 ~ -0.17)
Psychological	<0.001	-0.61 (-0.73 ~ -0.49)	0.022	-0.20 (-0.37 ~ -0.03)
Social	<0.001	-0.45 (-0.58 ~ -0.33)	0.005	-0.29 (-0.50 ~ -0.09)
Entertainment	<0.001	-0.27 (-0.37 ~ -0.17)	0.054	-0.15 (-0.29 ~ 0.00)

a Unadjusted model

b Adjusted for sex, residence, education level, marital status, employment status, economic status, smoking, drinking, sleep duration, exercise, history of psychiatric and physical illness, type of diabetes, complications, current medication plan, age, illness duration, and the scores of PHQ-9, GAD-7, ISI, DDS-17, and PSS.

## 4 Discussion

To our knowledge, this is the first study exploring the prevalence, correlates, and association of PLEs with QOL in patients with DM. The major findings are as follows: (1) The prevalence of any PLEs (74.75% vs. 49.67%), frequent PLEs (35.29% vs. 4.30%), and clinically relevant PLEs (13.85% vs. 0.66%) was consistently substantially higher in the DM group. The presence of diabetes was independently associated with a higher likelihood of PLEs; (2) Significant differences were found between diabetes patients with and without PLEs in terms of demographics, diabetes-related variables, mental health problems, and QOL. Age, smoking status, diabetes distress, perceived stress, and depressive symptoms were independently associated with PLEs; (3) Patients with PLEs demonstrated much worse QOL across multiple domains. The association of PLEs with impaired QOL remained significant after full adjustments.

### 4.1 Prevalence of PLEs among patients with DM

Consistent with our Hypothesis 1, we found a significantly higher prevalence of PLEs among patients with DM (up to 74.75%), a rate significantly higher than that observed in the Chinese general population, which ranged from 30% to 50% (36–38). Patients with DM were 1.48 times more likely to report concurrent PLEs than health control, after controlling for demographics including age, sex, and education years. This rate was similar to a recent finding by Slerus et al. (16), who also found patients with DM were 1.25 times more likely to have at least one PLE in the American population. Notably, the association between DM and the frequent and clinically relevant PLEs was even stronger, highlighting the vulnerability of this population.

### 4.2 Associated factors of PLEs among patients with DM

Our study represented the first investigation into the factors associated with PLEs in patients with DM. Consistent with our hypothesis 2, we found those with and without frequent PLEs differed significantly in demographic, diabetes-related variables, mental health, and QOL. In line with findings in the general population, we found younger age and smoking status were independently associated with PLEs in patients with DM. For example, Rep C et al. (19), reported that the prevalence of PLEs decreased across age from 34.7% in the 20–29 years age group, to 19.7% in the 70+ years age group. In a large study involving 34,653 participants, Mallet J et al. (39), found that 26.33% of nonsmokers reported at least 1 PLE, this prevalence was slightly higher in former smokers (27.48%) and rose as high as 39.09% in current smokers. All 22 PLEs had a higher prevalence in smokers than in former smokers or lifetime abstainers. A total of 8.56% of smokers reported at least 5 PLEs, compared to 3.42% in lifetime abstainers (AOR = 1.56; 95% CI, 1.32-1.84). Wang D et al. (40), also found that adolescents who smoked showed a higher prevalence of PLEs than in non-smoking samples. The above studies suggest that there was a significant association between smoking status and PLEs prevalence. Thus, anti-smoking measures in educational settings directed at both adolescents and their caregivers may decrease occurring rates of PLEs among adolescents.

Patients with PLEs reported higher levels of mental distress, including depression, anxiety, insomnia, and stress. Among them, stress and depression were independently related to PLEs. This finding aligned with those in the general population, which also revealed a strong and positive association between PLEs and other mental distress (10, 41, 42). The results called for comprehensive mental health assessments in DM patients who suffered from PLEs. Special attention should be paid to depressive symptoms and stress.

Interestingly, our study provided the first empirical evidence of the independent association between diabetes distress and PLEs, which, to our knowledge, has rarely been reported. Diabetes distress refers to the emotional burdens and worries specific to managing diabetes, encompassing feelings of frustration, burnout, and fear related to disease management and its complications (43). A recent meta-analysis suggested that half of the Chinese patients with DM suffered from diabetes distress (44). There were a few potential explanations. First, chronic stress from diabetes management can lead to dysregulation of the hypothalamic-pituitary-adrenal (HPA) axis, resulting in elevated cortisol levels that affect cognitive and perceptual processes associated with PLEs (45). Second, persistent emotional strain may exacerbate feelings of helplessness and cognitive distortions (46), increasing vulnerability to subclinical psychotic symptoms. Future studies were needed to replicate our findings and to verify the potential mechanism.

### 4.3 Association between PLEs and impaired QOL in patients with DM

Consistent with hypothesis 3, our study suggested that individuals with DM who reported PLEs demonstrated lower scores across all domains of QOL. The association between PLEs and QOL remained significant after adjusting for other diabetes-related and mental health problems. This relationship may be attributed to the pervasive impact of PLEs on daily functioning and mental health, which can exacerbate the challenges of managing a chronic condition like diabetes. PLEs may lead to increased stress, reduced motivation for self-care, and heightened feelings of isolation, all of which contribute to a diminished sense of well-being and overall life satisfaction (47, 48).

### 4.4 Strength and limitations

Our study has several limitations. First, the cross-sectional design precludes causal inferences, and the reliance on self-report measures is subject to recall and social desirability biases. Despite the excellent reliability and validity of the CAPE-15 demonstrated in previous studies, the use of self-reported questionnaires might increase the likelihood of false positives. Second, patients were recruited from two hospitals. our sampling strategy affects the generalizability of our findings. The use of non-probability methods—convenience sampling for patients in hospital settings and snowball sampling for community controls—is susceptible to selection bias and reduces comparability between the groups. Consequently, our findings may not be representative of the broader diabetes population and are most applicable to similar clinical settings. A further limitation is our inability to exclude participants with diagnosed psychotic disorders. While we statistically controlled for a self-reported general “history of psychiatric illness,” our questionnaire did not differentiate between specific diagnoses. Therefore, the potential inclusion of individuals with a clinical psychotic disorder may have inflated the reported prevalence of PLEs, conflating formal symptoms with the subclinical experiences of primary interest. The differential compensation between groups, though IRB-approved to account for participant burden, could have influenced participation patterns.Third, although our study provided a comprehensive assessment of demographic, diabetes-related variables, and mental distress, several well-established risk factors, such as childhood trauma, recent stressful life events, and biological markers like brain structure and HbA1c levels, were not measured. The link between diabetes and PLEs could be influenced by shared underlying liabilities, such as inflammatory pathways, as well as potential measurement overlap between PLEs and general affective distress. Finally, a methodological limitation is the absence of a formal *a priori* power analysis to determine the required sample size. However, for our primary analysis using logistic regression, A study by Bujang et al. recommends a minimum sample size of 500 for logistic regression in large population studies to ensure the resulting statistics are a close approximation of the true population parameters (49). Furthermore, for the linear regression, our sample size meets the criteria of 20 subjects per variable (24*20 = 480), which was proposed to derive reliable and valid estimates. Given that our sample size (816) exceeds these thresholds, we are confident it was sufficient for a stable and accurate regression analysis.

## 5 Conclusion

To conclude, our study highlights the high prevalence and strong, independent association of psychotic-like experiences with impaired QOL among patients with diabetes, calling for timely and regular assessments for PLEs. Targeted interventions addressing smoking, depressive symptoms, stress, and diabetes distress may help reduce PLEs and improve the quality of life for patients with diabetes.

## Data Availability

The original contributions presented in the study are included in the article/supplementary material. Further inquiries can be directed to the corresponding author.
